# Anorexia of Aging: Risk Factors, Consequences, and Potential Treatments

**DOI:** 10.3390/nu8020069

**Published:** 2016-01-27

**Authors:** Francesco Landi, Riccardo Calvani, Matteo Tosato, Anna Maria Martone, Elena Ortolani, Giulia Savera, Alex Sisto, Emanuele Marzetti

**Affiliations:** Department of Geriatrics, Neurosciences and Orthopedics, Catholic University of the Sacred Heart, Rome 00168, Italy; riccardo.calvani@gmail.com (R.C.); matteo.tosato@rm.unicatt.it (M.T.); annamariamartone@gmail.com (A.M.M.); eleort@gmail.com (E.O.); giulia.savera@libero.it (G.S.); alexsisto@gmail.com (A.S.); emarzetti@live.com (E.M.)

**Keywords:** nutrition, protein, ghrelin, geriatric syndrome, supplementation, frailty, sarcopenia, malnutrition, appetite, food intake

## Abstract

Older people frequently fail to ingest adequate amount of food to meet their essential energy and nutrient requirements. Anorexia of aging, defined by decrease in appetite and/or food intake in old age, is a major contributing factor to under-nutrition and adverse health outcomes in the geriatric population. This disorder is indeed highly prevalent and is recognized as an independent predictor of morbidity and mortality in different clinical settings. Even though anorexia is not an unavoidable consequence of aging, advancing age often promotes its development through various mechanisms. Age-related changes in life-style, disease conditions, as well as social and environmental factors have the potential to directly affect dietary behaviors and nutritional status. In spite of their importance, problems related to food intake and, more generally, nutritional status are seldom attended to in clinical practice. While this may be the result of an “ageist” approach, it should be acknowledged that simple interventions, such as oral nutritional supplementation or modified diets, could meaningfully improve the health status and quality of life of older persons.

## 1. Introduction

Anorexia of aging, defined as the loss of appetite and/or decreased food intake in late life, is a notable paradigm of geriatric syndromes. This expression was coined because the multifaceted clinical conditions that are common among frail older persons are not easily grouped into specific diseases or “traditional” syndrome categories. Many of such clinical conditions are highly prevalent and related with many comorbidities and adverse outcomes, comprising disability and poor quality of life. Anorexia of aging is indeed associated with many of the syndromes and effects that occur when the accumulation of health impairments in multiple systems combine to make older persons more vulnerable to internal and/or external stressors.

**Figure 1:**
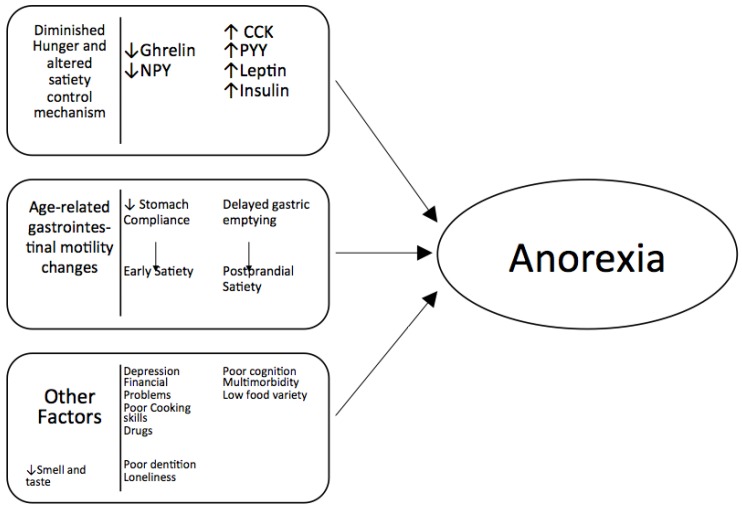
Major mechanisms involved in the development of anorexia of aging.

The regulation of appetite, particularly when deficient, is the key to understanding the pathogenesis of anorexia of aging. Food intake is controlled through highly complex processes, with fail-safe mechanisms in place to guarantee that the feeding drive remains unimpaired. To simplify, a central feeding pathway that is restrained by peripheral satiation signals regulates food ingestion. The central feeding system collects supplementary feedback from peripheral fat cells, specific nutrients, and circulating hormones. The multi-level modifications of this system during the aging process result in the “physiologic” anorexia of aging (Figure 1).

Complex mechanisms are involved in the age-related deterioration of specific activities in certain brain areas, such as the hypothalamus, in responses to peripheral stimuli (e.g., circulating hormones, adipokines, nutrients) [1,2,3]. While an exhaustive description of these processes is beyond the scope of this review, several factors that may contribute to the onset of anorexia of aging warrant a brief discussion (Figure 1).

## 2. Mechanisms of Anorexia of Aging

### 2.1. Smell and Taste

Smell and taste play an important role in making eating and drinking enjoyable. The sense of smell and taste decreases with age, although likely at differing rates. This contributes to diminished food intake in old age and also has a negative impact on the type of food ingested, typically resulting in a less varied and more monotonous diet. The number of taste buds also decreases during the aging process and the remaining buds start to be atrophic. Diseases, medications, smoking, and some environmental exposures may worsen the changes observed in the number and functionality of taste buds. Older persons frequently lose salty and sweet tastes first. Hence, some foods lack the taste to satisfy the appetite, with the consequence that older people might choose a more tasteful, yet, unhealthy diet. Finally, the decline in saliva secretion may reduce the ability to dissolve foods and limit their interaction with taste receptor cells on the tongue [4,5].

### 2.2. Hormones

*Ghrelin*, or the “hunger hormone”, is the only peripheral hormone identified to stimulate hunger. It is released in a pulsatile fashion by ghrelin cells embedded in the gastrointestinal mucosa placed in the stomach. Little evidence is available on how ghrelin dynamics change during the aging process. Nevertheless, it seems that a concomitant increase in circulating leptin and insulin may be correlated with lower sensitivity to ghrelin in older adults [5,6,7,8].

Similar to ghrelin, modifications in the dynamics of *cholecystokinin* (CCK) have been observed in older adults. CCK is the prototype of satiety hormones and is released by the proximal small intestine in response to the delivery of nutrients, mainly proteins and lipids, from the antrum [5,6,7,8]. Some observations suggest a potential role of modified CCK dynamics in the cause of anorexia of aging. Some other studies have also demonstrated an increase in serum concentrations of *peptide YY* (PYY) in the late postprandial phase in older persons relative to young controls. High postprandial PYY levels may hinder the desire for a second meal, leading to longer fasting times. Consequently, the combined actions of CCK and PYY convey important anorexigenic signals to the hypothalamus [5,6,7,8].

*Leptin* is an additional hormone that has been involved in the pathogenesis of anorexia of aging. High circulating levels of leptin are expected to play an important role in the postprandial pathway of signals in anorexia of aging [5,6,7,8]. Finally, the aging process is accompanied by an increase in fasting and post-prandial plasma insulin concentrations. *Insulin*, the master regulator of glucose metabolism, also acts as a satiety hormone. Indeed, the reduced glucose tolerance and elevated levels of insulin observed during aging may accelerate the development of anorexia. This action of insulin is performed indirectly by enhancing the anorexigenic signal of leptin to the hypothalamus and hindering the ghrelin stimulus [5,6,7,8].

### 2.3. Gastrointestinal Function

Abnormalities in gastric motility may cause early satiation correlated to reduced fundus compliance. In older persons, decreased secretion of nitric oxide has been described at the level of the fundus, which results in loss of gastric compliance and more rapid antral filling. Moreover, delayed gastric emptying may be responsible for protracted postprandial satiety. Slower gastric emptying in older persons may be associated with reduced digestive ability in the stomach and a primitive age-related failure of gastric motility. Chronic gastritis and some drugs (e.g., proton-pomp inhibitors) may cause hypochlorhydria, which further delays gastric emptying [5,9]. A slower gastric emptying may decrease the appetite and the food intake by enhancing and prolonging antral distension, as well as modifying the small intestine satiety signals.

### 2.4. Inflammation

Chronic low-grade inflammation, a hallmark of the aging process, may modify the response of target brain areas to peripheral stimuli. Circulating levels of interleukin (IL) 1, IL6 and tumor necrosis factor alpha (TNF-α) are typically higher in older adults independent of specific diseases or multimorbidity. Such cytokines reduce food intake and, hence, body weight by several means, contributing to delayed gastric emptying and clampdown of small intestinal motility. These cytokines directly stimulate leptin mRNA expression and enhance circulating leptin levels, too [10,11]. Besides their direct effects on leptin, pro-inflammatory cytokines also stimulate the production of hypothalamic corticotropin releasing factor (CRF), a mediator of the anorexigenic effect of leptin [10,12].

## 3. Risk Factors for Anorexia of Aging

There are many risk factors theoretically related with this syndrome that need to be assessed [13]. Among the described factors are physical function impairment, social and environmental conditions, acute and chronic diseases, and treatments.

### 3.1. Physical Factors

**Figure 2:**
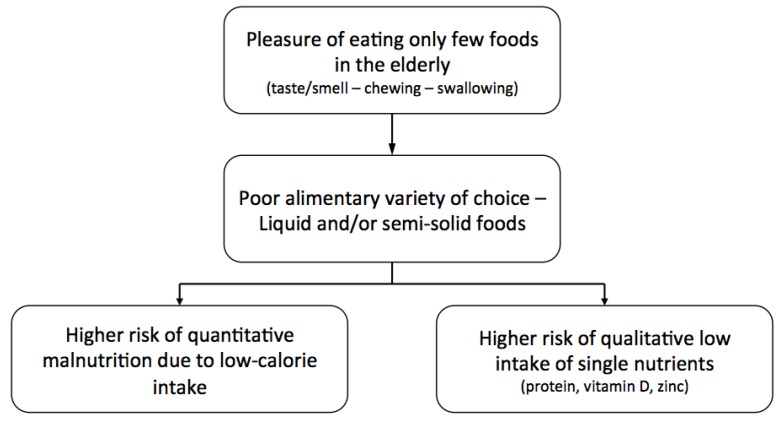
Anorexia of aging and risk of malnutrition.

Functional impairments in the basic and instrumental activities of daily living (ADL and IADL) are related with reduced food intake and loss of appetite. It is possible that physical impairment causes mobility limitations that, in turn, could be responsible for anorexia through multiple mechanisms. In particular, problems in eating by oneself, difficulty in getting foods, and lack of cooking skills are relevant risk factors for anorexia of aging. Functional deficiencies and sensory impairments—hearing and vision—may also interfere with the ability of older persons to shop for, prepare, and consume food. Additional physical factors, such as poor dentition and ill-fitting dentures, may limit the type and quantity of food consumed. These conditions are correlated with chewing problems that can lead to poor nutritional status and modifications in the type and quality of nutrient intake. The presence of chewing problems is associated with lower intake of specific nutrients, including fibers, vitamins, calcium, and proteins, and with a higher intake of fats and cholesterol (Figure 2) [13,14].

### 3.2. Medical Factors

Specific medical conditions in older persons, such as gastrointestinal diseases, malabsorption syndromes, acute and chronic infections, and hypermetabolism (e.g., hyperthyroidism), often cause anorexia and micronutrient deficiencies, in the face of increased energy requirements [15]. Furthermore, older adults frequently suffer from diseases that modify the appetite and cause malabsorption or increased metabolism. For example, congestive heart failure (CHF), chronic obstructive pulmonary disease (COPD) and Parkinson’s disease are frequently associated with both anorexia and increased energy expenditure [15].

Depression is one of the most common psychological disorders among older people and is often associated with loss of appetite. Among those diagnosed with depression, older adults seem to suffer more severe appetite and weight loss than younger persons. Depressed older individuals have numerous symptoms and signs that can contribute to anorexia and weight loss, including weakness, stomach aches, nausea, and diarrhea. Loss of appetite and reduced food intake are also frequently observed in older adults with cognitive impairment, especially in the later stages of the condition.

### 3.3. Medications

Older persons typically take many prescription as well as over-the-counter medications, a number of which can cause malabsorption, gastrointestinal disorders, loss of appetite and ultimately reduced food intake [4,15,16]. The risk of drug-induced anorexia is further increased by polypharmacy, due to the enhanced odds of drug–drug interactions and gastrointestinal problems [17].

### 3.4. Social Factors

The main social factor that contributes to decrease appetite and food intake in old age is socio-economic inequality. Social isolation is also certainly one important factor contributing to the onset of anorexia of aging. Living alone is indeed associated with decreased appetite and energy intake [18]. In particular, failure by a long-term care facility to pay attention to residents’ food preferences and to adequately stimulate a favorable environment to eat are important factors related to the loss of appetite and reduced food intake among older residents. In fact, among institutionalized older individuals, anorexia and subsequent unintentional weight loss may be the consequence of monotony and repetitiousness of daily foods.

## 4. Assessment of Anorexia of Aging

Validated screening tools are available to identify older persons with anorexia or at risk of developing it. Visual analogue scales can reveal decreased spontaneous food intake and specific questionnaires can document nutrient intakes lower than 70% of estimated needs. The Simplified Nutritional Assessment Questionnaire (SNAQ) is a simple screening tool with good predictive ability for future weight loss and protein-energy malnutrition [19,20]. Moreover, the section AC/S-12 of the Functional Assessment of Anorexia/Cachexia Therapy (FAACT) questionnaire may be used to recognize anorexia-related symptoms and grading the severity of each of them scoring from 0 (worse score) to 4 (better score). A score of 24 has been proposed as diagnostic for anorexia [21,22].

**Figure 3:**
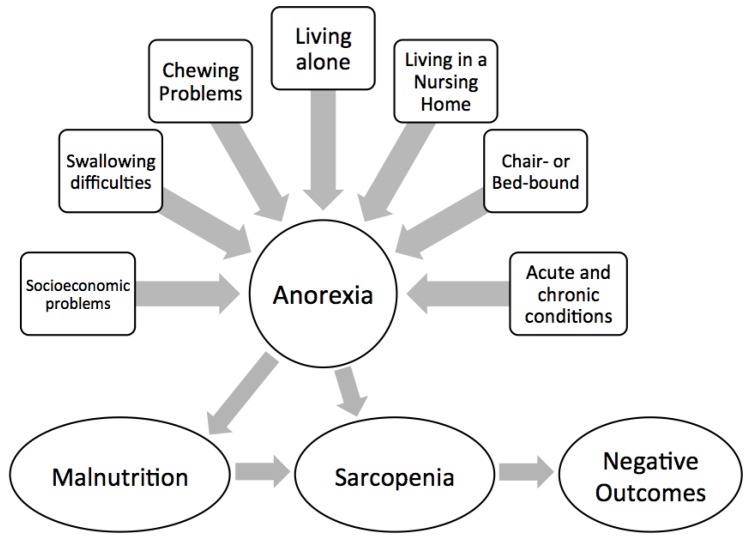
Risk factors for anorexia of aging and negative outcomes.

**Figure 4:**
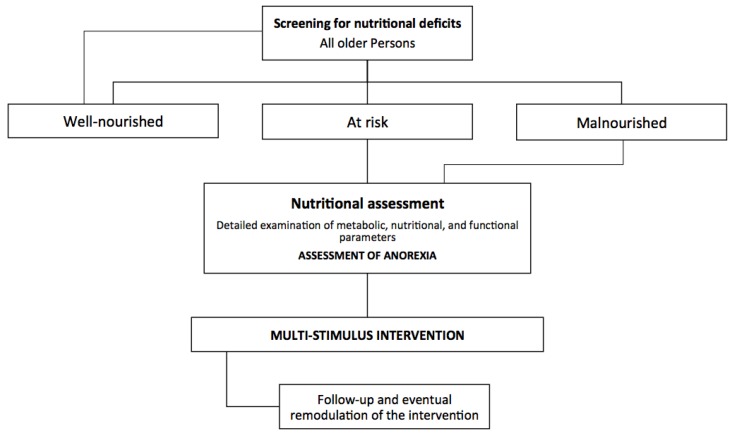
Assessment of anorexia of aging and malnutrition.

Multidimensional programs that aim to identify and address risk factors for anorexia of aging are particularly interesting (Figure 3). The first step in the management of anorexia is the identification of persons at risk of developing the condition by using second- and third-generation geriatric assessment tools (e.g., MDS-*inter*RAI tools) [23]. The *inter*RAI system is comprised of a suite of comprehensive geriatric assessment instruments that are able to identify clinical, psychological, socioeconomic, and environmental conditions across different healthcare settings [23]. Modifications in feeding patterns may be sufficient in milder cases, whereas the correction of specific deficiencies and/or a systematic dietary revision may be needed in more advanced cases. When a specific and individualized intervention is established, follow-up assessments should be performed to estimate the effectiveness of the treatment plan (Figure 4). The *inter*RAI system is particularly suited for this purpose as it permits encompassing individualized nutritional interventions across different healthcare settings.

## 5. Consequences of Anorexia of Aging

Strong evidence suggests that anorexia of aging is related to adverse health outcomes. In particular, anorexia of aging is the substrate for overall or selective malnutrition, sarcopenia, and physical frailty (Figure 2).

### 5.1. Malnutrition

Anorexia is associated with a higher risk of quantitative malnutrition (for example, protein-energy malnutrition) due to inadequate overall nutrient intake. Particularly in the early stages, anorexia increases the risk of qualitative malnutrition, due to suboptimal intake of single nutrients, like proteins and vitamins [21]. Studies have demonstrated that selective malnutrition is also related with the development of sarcopenia and several other negative health outcomes, comprising morbidity and mortality [24,25]. This association may offer a plausible explanation for the frequent association of anorexia with poor endurance, slow gait and reduced mobility [26].

### 5.2. Frailty and Sarcopenia

Inadequate food intake frequently results in reduced physical activity and declining muscle mass and strength [27]. Studies conducted in older community-dwellers have shown that anorexia was associated with impaired physical performance and significantly increased the risk of incident disability, after controlling for potential confounders [28]. Four-meter walk speed, short physical performance battery (SPPB), handgrip strength, and ADL score were worse among older adults presenting with anorexia and weight loss. This indicates that anorexia of aging is directly involved in the development of frailty [29,30] (Figure 3).

Notably, insufficient consumption of leucine and/or Vitamin D, which may occur during anorexia, appears to be causally linked with the development of sarcopenia and frailty [27,30]. Supplementation with essential amino acids, that could counteract such a nutritional deficiency, has recently been shown to improve muscle mass in old age [31,32].

The current recommended dietary allowance (RDA) for protein is 0.8 g/kg/day. However, there is agreement that protein intake in older people should be brought up to 1.0–1.3 g/kg/day. Furthermore, it is important to highlight that protein should be consumed in a pattern spread out through the day (approximately 30 g at each meal for a 70-kg person) in order optimize the muscular anabolic response [33,34]. Finally, Vitamin D supplementation (800 UI/day) increases the number and cross-sectional area of type II muscle fibers (which are typically lost in sarcopenic persons) [35]. This adaptation has been demonstrated to increase muscle mass and strength and at the same time reduces the risk of falls and injuries.

### 5.3. Mortality

Assessing the impact of anorexia on survival among older adults is an important and complex issue [36]. Studies conducted in a sample of community-living persons aged 65+ years revealed that anorexia and unintentional weight loss are powerful risk factors of mortality, independent of age, gender and other potential confounders [37]. The impact of anorexia on mortality has also been established by results from the “Un Link Informatico sui Servizi Sanitari esistenti per l'anziano—a computerized network on health care services for older people” (ULISSE) project, a study designed to assess the quality of care in nursing home residents [13]. In this study, a direct association was determined between mortality and anorexia in 1904 elderly residents of both genders. In particular, subjects with anorexia had an almost two-fold higher risk of death for all causes compared with subjects without anorexia.

## 6. Treatment of Anorexia of Aging

The prevention and treatment of anorexia may be accomplished through multi-stimulus interventions, including food manipulation, correction of environmental and pharmacological risk factors, and treatment of underlying medical causes [15,26,38].

### 6.1. Food Manipulation

This approach involves the enhancement of food texture and palatability, flavor improvement, provision of dietary variety, and feeding assistance as needed.

### 6.2. Environmental Adaptation

This intervention is aimed at preventing social isolation and endorsing conviviality, particularly in nursing home residents.

### 6.3. Medication

The evaluation of pharmacological therapies is required to identify drugs that may decrease appetite and/or favor weight loss. The most frequently prescribed drugs that may hinder appetite comprise: (1) cardiovascular drugs such as digoxin, amiodarone and spironolactone; (2) psychiatric drugs such as phenotiazines, lithium, amitriptyline, fluoxetine and other selective serotonin reuptake inhibitors; and (3) anti-rheumatic drugs such as non-steroidal anti-inflammatory agents. Other medications can contribute to weight loss by causing malabsorption (e.g., laxatives) or increasing metabolism (e.g., theophylline).

### 6.4. Medical Diagnoses

All possible medical causes that can contribute to weight loss need to be evaluated and specifically addressed. These comprise swallowing disorders (e.g., dry mouth, tooth loss, lesions or sores in the mouth), dyspepsia (e.g., gastritis and ulcers), malabsorption syndromes (e.g., bacterial overgrowth, gluten enteropathy, pancreatic insufficiency), neurological causes (e.g., stroke with residual swallowing deficits), endocrine disorders (e.g., hypercalcemia), psychiatric disorders (e.g., depression, delirium), respiratory diseases (e.g., COPD), and cardiovascular diseases (e.g., CHF).

### 6.5. Specific Treatments

Presently, no specific therapeutic agents have shown to be clearly effective in treating anorexia of aging. Nutritional supplementations do not directly cure anorexia of aging but only its consequences, such as weight loss and energy-protein malnutrition. A small number of studies have demonstrated positive effects from energy supplementation in malnourished older adults. Nevertheless, the heterogeneity of the supplementation protocols adopted hinders their applicability to routine patient care. The only clear evidence is currently limited to protein supplementation. According to the position paper from the PROT-AGE Study Group [39], a daily intake in the range of at least 1.0–1.2 g protein per kilogram of body weight is required to reduce the loss of muscle mass and strength and prevent the development of frailty.

Several medications have been examined to stimulate appetite in older adults, but none of them is actually recommended in routine clinical practice [15,26,38]. Corticosteroids increase body weight, primarily through increases in fat mass and fluid retention. Growth hormone also produces weight gain in malnourished older persons, but does not improve any physical and functional outcomes. Anabolic steroids (e.g., testosterone and oxandrolone) have been tested in older people with some positive results, but they have numerous adverse effects, such as cardiovascular events and liver dysfunction. Metoclopramide may control the symptoms correlated to the early satiety; however, its long-term use is associated with important negative side effects, namely extra-pyramidal symptoms. Similarly, other appetite-stimulating medications (e.g., megesterol, meclobemide, tetrahydrocannabinol, cyproheptadine, CCK antagonists such as loxiglumide) have been associated with numerous side effects, including delirium and abdominal symptoms. For these reasons, they are of limited benefit in clinical practice.

## 7. Conclusions

Anorexia of aging, with its high prevalence and negative impact on quality of life, morbidity and mortality, represents one of the major challenges of geriatric medicine. Anorexia needs to be considered an important indicator of significant disorder of energy metabolism during the aging process. For this reason, a more thorough comprehension of the mechanisms of energy metabolism in later life is necessary to develop effective preventive and therapeutic interventions.

One of the most important aims in the care of older adults is the enhancement of their nutritional status. In this respect, the first step is the identification of perosns that are at risk of anorexia of aging by using second- and third-generation geriatric assessment tools. Then, potentially reversible factors that promote loss of appetite and diminish food intake should be eliminated in order to prevent the development of anorexia [40]. Specific individualized care plans should consequently be implemented to guarantee the provision of adequate amounts of food and limit weight loss. Finally, multi-stimulus interventions and specific strategies, including food texture adjustments, flavor enhancements and feeding assistance, may be effective in the management of anorexia in frail and institutionalized older people.
